# Control of neural systems at multiple scales using model-free, deep reinforcement learning

**DOI:** 10.1038/s41598-018-29134-x

**Published:** 2018-07-16

**Authors:** B. A. Mitchell, L. R. Petzold

**Affiliations:** 1 Department of Computer Science, University of California, Santa Barbara, USA; 2 Department of Mechanical Engineering, University of California, Santa Barbara, USA

## Abstract

Recent improvements in hardware and data collection have lowered the barrier to practical neural control. Most of the current contributions to the field have focus on model-based control, however, models of neural systems are quite complex and difficult to design. To circumvent these issues, we adapt a model-free method from the reinforcement learning literature, Deep Deterministic Policy Gradients (DDPG). Model-free reinforcement learning presents an attractive framework because of the flexibility it offers, allowing the user to avoid modeling system dynamics. We make use of this feature by applying DDPG to models of low-level and high-level neural dynamics. We show that while model-free, DDPG is able to solve more difficult problems than can be solved by current methods. These problems include the induction of global synchrony by entrainment of weakly coupled oscillators and the control of trajectories through a latent phase space of an underactuated network of neurons. While this work has been performed on simulated systems, it suggests that advances in modern reinforcement learning may enable the solution of fundamental problems in neural control and movement towards more complex objectives in real systems.

## Introduction

With the advent of deep learning and deep neural networks, reinforcement learning has rapidly advanced beyond methods in classical neuro-dynamic programming by the publication of the Deep Q-Network (DQN)^1^. This approach to Q-learning relies on convolutional neural network approximations of both the policy as well as the value (Q) function and was used to allow a computer to better learn to play Atari games. Since its publication, deep Q-learning has been applied to a number of different physical problems. Particularly relevant to neural control is the extension of DQN to problems with continuous state and action spaces by the Deep Deterministic Policy Gradient (DDPG) method^2^.

At the same time, application of deep reinforcement learning to neural control has not yet been reported and in general, the application of deep reinforcement learning for control of biological systems has not been reported either. There are many reasons for this, but perhaps the most important and serious hurdle involves the difficulty in constructing accurate models of complex biological systems amenable to control. This is not to say that good models don’t exist: in fact, a number of approaches have been tried including dynamical systems^3–10^ and statistical approaches using a point-process model of past inputs and spikes^11–15^. It is certainly possible to incorporate a deep neural network into these existing approaches (e.g. using a deep neural network to fit the rate of the point process, as in^16^). From either a dynamical systems or a statistical perspective though, networks of neurons are difficult to model in that the modeling process requires significant interdisciplinary insight. In addition to the non-linearity and stochasticity of these systems, there is the added difficulty of selecting the scale at which to model these systems. Behavior can be strongly influenced by single protein dynamics as well as small and large neural population dynamics and the selection of the scale at which to model these systems can be non-trivial. Moreover, the construction of a model for a single scale is typically useless when trying to model events at other scales.

Due to the current complexities and limitations of models of actual neural systems, we have chosen to pursue a control strategy that is model-free (i.e. DDPG). This framework frees us from the need to model the state transition dynamics at all. Such an approach may be useful for fields including translational research whose goal is to generate medical therapies: in these areas, understanding the dynamics of the pathological system is often thought of as a prerequisite for developing a therapy. We argue that our results indicate that this may not always be necessary. These results would also allow researchers to easily adapt our control system to changes in system dynamics. While these advantages are significant, there are additional challenges posed by a model-free strategy that are not present in a model-based control strategy. For example, many have criticized model-free methods for their poor sample complexity^17^, claiming that this limits their applicability to real systems^18^. We observe that for the synthetic neural systems used in this paper, a Kuramoto Model (KM) of synchronized oscillators and a network of Stochastic, Leaky Integrate and Fire (SLIF) neurons, the sample complexity was reasonable given proper definition of the reward function.

This point raises another of the difficulties involved in model-free control, namely the proper design of a reward function. The reward function must be precisely designed so as to inform the policy as to the goals of the problem, but the state-action space with non-zero reward must be reachable in a reasonable amount of time from the initial conditions. This relationship requires proper coordination between the design of the reward function, the definition of the state space, and the use of exploration during fitting of the policy and value functions. For SLIF control (both fully- and under-actuated problems), we find that simple rewards are sufficient to achieve good performance. Though for control of the Kuramoto Model, we make use of shaped rewards. These reward functions allow for the decomposition of complex tasks into simpler ones with smaller rewards being given for partial solutions of the full objective. In this case, the full objective we consider is the synchronization of a network of weakly coupled oscillators with the intermediate objective of synchronization of each oscillator in the network with a reference oscillator (i.e. entrainment of the network). In addition, we make use of an Ornstein-Uhlenbeck process for the exploration of the state-action space. This choice was motivated both by the previous use of this process with DDPG as well as the neurological significance of this process. For example, under certain conditions it is thought that the evolution of synaptic spine strength follows an Ornstein-Uhlenbeck process^19,20^. Since the connection strength between two cells is roughly proportional to the sizes of the spines on the synapes between them, exploration of the space of spine sizes can be interpreted as exporing the space of possible Extra-cellular Post-Synaptic Potentials that may be generated by a given pre-synaptic cell. Further, Ornstein-Uhlenbeck noise has also recently been used as a model of large-scale neural dynamics^21^ during a decision making task.

We claim that DDPG, and deep reinforcement learning in general, has considerable promise for general purpose neural control. We begin this paper with sufficient background on deep reinforcement learning and neural control to allow the reader to judge this claim. We then describe the models that are the object of the control and the manner in which the problem is formulated for each model system. For each system, the objective is slightly different, the analysis must be slightly different. In general, we show empirically that we are able to achieve a wide array of different objectives on each system in a sample efficient and scalable manner.

## Background

### Background on Neural Control

Past approaches to neural control have focused on dynamical systems-based formulations of the control problem, where methods have been designed to control a single model of neural dynamics^3–10^. Specifically, in^9^ a method is developed for directly optimizing oscillator coupling strength to impose synchronization on the system. While an interesting approach, this method is unlikely to directly lead to algorithms that can be implemented on real neural systems because the coupling weights between oscillators can be difficult to perturb. The authors of^7,8^ use a different model for system inputs than we do: they assume that a common forcing input is applied to all oscillators. In addition, these control models are open-loop strategies: the authors give a number of motivations for this approach, stating that system dynamics and network connectivity is difficult to estimate and state information may be unavailable. For large scale neural systems, while the dynamics are largely unknown, high spatial resolution state observations can be obtained using functional Magnetic Resonance Imaging (fMRI) and detailed network connectivity can be obtained using Diffusion Tensor MRI (DT-MRI). More importantly though, while their results show that this forcing term can lower the minimum coupling strength at which synchronization occurs, there is still a lower-bound below which synchronization no longer occurs, even in the presence of forcing. Our claim is that DDPG is able to synchronize a collection of oscillators past this lower bound and we compare DDPG with this method in the results section.

Other dynamical systems approaches include^3–6,10^, where the authors employ phase reduction methods to models of neural oscillations to better understand how one might desynchronize^3–6^ or synchronize^10^ the oscillators (though in^10^, the problem of control was mentioned as a potential application, but not studied). These methods involve a search for a reduced set of phases (often a single scalar), whose dynamics well-characterize the oscillatory behavior of each oscillator in the network. The study of the movement of these oscillators towards (synchronization) or away from (desynchronization) these limit cycles is the focus of these papers. These methods are different from ours, not only in the fact that they are model-based, but also because they work primarily with low-dimensional dynamics. In particular, it is not clear how these methods would apply to similar control problems where the dynamics are high-dimensional.

Statistical approaches to control exist with both model-based^11,12^ and model-free variants^13,14^ having been attempted. In particular, the authors of^12^ show how a controller based on a Generalized Linear Model (GLM) can be used to produce target spike sequences in an underactuated setting. While impressive, it is not clear how these results extend to the ability to generate or manipulate biologically meaningful states. Relying on correlated activity between neurons, we show how principal component trajectories can be induced in an underactuated setting. Principal Component Analysis (PCA) has been used in a number of experiments attempting to reduce neural dynamics to a lower dimensional manifold^15,22–24^. The dynamics in the phase space defined by a few principal components has been related to simple behaviors, for example, reaching to a specific point in space results in a characteristic trajectory of primary motor cortex in the phase space of its first three principal components. And while the authors of^13,14^ also employ model-free reinforcement learning for neural control, they attempt to solve a different problem from the ones we consider in this paper, that of the desynchronization of a network of synchronized, coupled oscillators. The advancements of deep reinforcement learning since the publication of these methods suggest that model-free reinforcement learning might be applied to more difficult, poorly understood problems.

### Deep Q-Learning

The method used in this paper is a variant of an approach towards optimal control problems called Q-learning. The goal of Q-learning is to estimate an action-value function (i.e. a Q-function) which is a measure of the expected future rewards, given a fixed policy, *μ*(*a*_*t*_|*s*_*t*_). This Q-function may then be optimized by finding a policy that maximizes this quantity; this is the primary use we make of the Q-function in this paper. One of the particularly attractive features of Q-learning is that it is a model-free control strategy. This means that no explicit model of the state-transition dynamics is estimated during computation of the policy. Thus for Q-learning, particular importance is placed on finding a good estimator of the Q-function, and in this paper, we use a deep neural network to estimate Q.

The deep reinforcement learning literature typically begins by assuming that the system under consideration can be modeled with a Markov Decision Process (MDP). In a MDP, we have a state space $S\subseteq {R}^{n}$, an action space $A\subseteq {R}^{m}$, an initial state distribution *p*(*s*_1_), transition dynamics *p*(*s*_*t* + 1_|*s*_*t*_, *a*_*t*_), and a reward function *r*(*s*_*t*_, *a*_*t*_). The MDP framework allows for a convenient representation of the Q-function. The Q-function can be written as

1 $${Q}^{\mu }({a}_{t},{s}_{t})={{\rm{E}}}_{{r}_{i\ge t},{s}_{t > t},{a}_{i > t}\sim \mu }[{r}_{t}|{a}_{t},{s}_{t}],$$

where ${r}_{t}={\sum }_{i=t}^{T}{\gamma }^{i-t}r({s}_{i},{a}_{i})$, $\gamma \in R$ is the discounting factor, and $a\sim \mu$ indicates the sampling of an action from the policy. Under an MDP, it may be written as

2 $${Q}^{\mu }({a}_{t},{s}_{t})={{\rm{E}}}_{{r}_{t},{s}_{t+1}}[r({s}_{t},{a}_{t})+\gamma {{\rm{E}}}_{{a}_{t+1}\sim \mu }[{Q}^{\mu }({a}_{t+1},{s}_{t+1})],$$

where this equation is known as the Bellman equation. A common approach to estimating the optimal policy from the Bellman equation is to estimate *μ* in a greedy fashion by computing $\mu ({a}_{t}|{s}_{t})={\rm{\arg }}\,{a}_{t}\,{\rm{\max }}\,{Q}^{\mu }({a}_{t},{s}_{t})$. This is the approach taken in^1^, where a deep convolutional network is used to approximate the Q-function.

**Figure 1:**
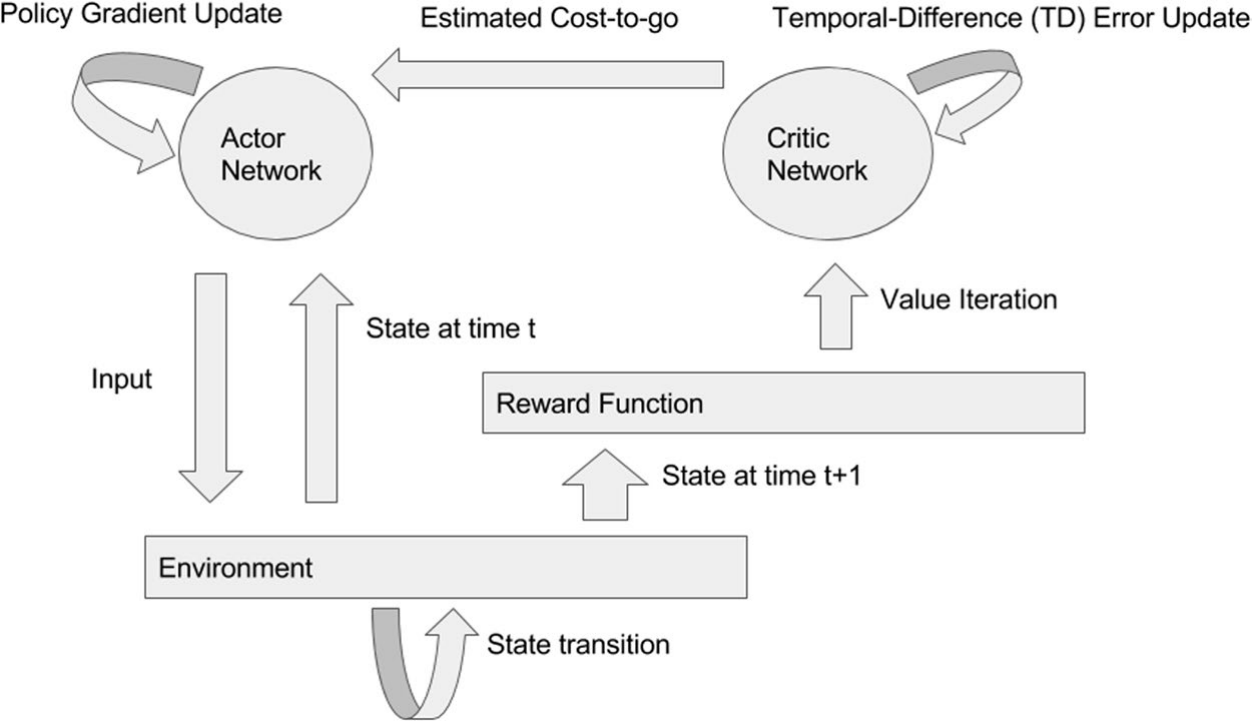
Diagram of the DDPG algorithm. The core components of this algorithm are the actor network (i.e. the policy), the critic network (i.e. the value function), and the environment (in this paper, either the SLIF or Kuramoto model). The environment accepts inputs from the actor network and produces a state transition. The new state and action used to generate it are passed to the reward function. The reward function passes an update to the critic network, which estimates the value of the state-action pair at the current timestep. The parameters of the actor network are updated using the new policy gradient and the parameters of the critic network are updated using the new TD-error.

While this approach has been extremely successful on a number of different problems, it is impractical for large, continuous action spaces. Lillicrap *et al*.^2^ proposed to modify this approach by incorporating a Deterministic Policy Gradient (DPG) (graphical description in Fig. 1)^25^. In this case, *μ* is assumed to be a deterministic function and the parameters of the policy are updated along the following gradient

3 $${\nabla }_{{\theta }^{\mu }}J\approx {{\rm{E}}}_{{s}_{t}\sim {\rho }^{\beta }}[{\nabla }_{{\theta }^{\mu }}{Q}^{\mu }(a,s|{\theta }^{Q}{)|}_{s={s}_{t},a=\mu (s{(}_{t}|{\theta }^{\mu }))}],$$

4 $${\nabla }_{{\theta }^{\mu }}J\approx {{\rm{E}}}_{{s}_{t}\sim {\rho }^{\beta }}[{\nabla }_{a}{Q}^{\mu }(a,s|{\theta }^{Q}{)|}_{s={s}_{t},a=\mu ({s}_{t}|{\theta }^{\mu })}{\nabla }_{{\theta }^{\mu }}\mu {(s|{\theta }^{\mu })}_{s={s}_{t}}],$$

where *ρ*^*β*^ is a behavior policy potentially distinct from *μ*, and *θ*^*μ*^ and *θ*^*Q*^ are the parameters of the policy and the value function respectively. The utility of an off-policy algorithm in the context of neural control (and biological control in general) is significant. For the simulated systems considered in this paper, being able to estimate the policy gradient in an off-policy fashion allows us to avoid resampling states with every step of the gradient optimization and to reuse past samples (i.e. experience replay). In the setting of general biological control, sampling from a policy could involve a procedure with a deleterious cumulative effect (e.g. stimulating a collection of neurons as in deep brain stimulation) and the ability to minimize the number of times this is performed could be quite valuable.

The parameters *θ*^*Q*^ are updated along the gradient of a separate loss function

5 $$L({\theta }^{Q})={{\rm{E}}}_{{s}_{t}\sim {\rho }^{\beta },{a}_{t}\sim \beta }[({Q}^{\mu }({a}_{t},{s}_{t}|{\theta }^{Q})-{y}_{t}{)}^{2}],$$

where *y*_*t*_ is given by

6 $${y}_{t}=r({s}_{t},{a}_{t})+\gamma {Q}^{\mu }({a}_{t+1},{s}_{t+1}|{\theta }^{Q}\mathrm{).}$$

The loss function *L*(*θ*^*Q*^) is known as the temporal difference error (TD-error) and there is a long literature about its properties and potential applications^26^. The gradients, ▽_*θμ*_*J* and ▽_*θQ*_*L*, are used to alternately perform updates of *θ*^*μ*^ and *θ*^*Q*^ respectively. This approach is termed an actor-critic method, where the actor, *μ*(*a*_*t*_|*s*_*t*_, *θ*^*μ*^), is used to propose actions and the critic, *Q*^*μ*^(*a*_*t*_, *s*_*t*_|*θ*^*Q*^), declares the value of those actions.

The creators of DDPG demonstrate its use on a number of physical control problems, but the application of this method or other deep reinforcement learning methods to problems in biology is lacking. In the next section, we address this problem by showing how DDPG can be used for neural control of a network of SLIF neurons and a network of weakly coupled oscillators.

## Results

### Network of Stochastic, Leaky Integrate and Fire Neurons

A great deal has been published about simulated networks of single neurons, making this class of models an important benchmark for our system. We chose the Stochastic, Leaky Integrate and Fire (SLIF) model for the validation of our system, and connected these neurons in a random, directed network. The SLIF model is given by

7 $$\frac{d{V}_{i}(t)}{dt}=\frac{-1}{{\tau }_{v}}{V}_{i}(t)+\frac{1}{C}(b{u}_{j}(t)+\sum _{j}{A}_{i,j}{I}_{syn,j}(t))+\eta {e}_{i}(t),$$

8 $${I}_{syn,j}(t)=-\,{g}_{syn,j}(t)({V}_{i}(t)-{E}_{syn}),$$

9 $${g}_{syn,j}(t)=\bar{g}\frac{t-{t}_{s}}{{\tau }_{s}}\exp (\frac{-(t-{t}_{s})}{{\tau }_{s}}),$$

where *τ*_*v*_ is the membrane time constant, *C* is the membrane capacitance, *e*_*i*_(*t*) is the standard Gaussian white noise of the *i*’th cell, *η* denotes the standard deviation of this noise, ${u}_{i}(t)\in {R}^{S}$ is the extrinsic control input to the *i*’th cell, $b\in {R}^{1\times S}$ denotes the influence of the input on the neuron, *I*_*syn*,*j*_(*t*) is the synaptic current coming from the *j*’th neuron firing an action potential at time *t*_*s*_, *A*_*i,j*_ is the weight of the connection between the *i*’th and *j*’th cells, *E*_*syn*_ is the reversal potential of the synapse, $\bar{g}$ models the constant synaptic conductance, and *τ*_*s*_ determines the decay of the synaptic current as time elapses from the incoming spike at *t*_*s*_. For these experiments, we assume the existence of only a single type of cell, that is, a generic excitatory cell. All values of model parameters used to generate the results shown in this paper are given in the Methods section.

### Fully-Actuated Network Control

For the first application of DDPG, we solve the toy problem of inducing an arbitrary spike train in each neuron of the network, where each target spike train is drawn at random from a Poisson distribution. A model-free controller of this system must learn a number of key tasks: to depolarize each neuron at the appropriate times, to hyperpolarize the neuron at other times, and to generate inputs to each cell sufficient to wash out the effects of neighboring neurons. The simple reward function

10 $$r({s}_{i,t},{a}_{i,t},{s}_{i,t+1})=\{\begin{array}{cc}1, & {\rm{i}}{\rm{f}}\,\,{s}_{i,t+1}={h}_{i,t+1},{s}_{i,t+1}\ne 0\\ -1, & {\rm{i}}{\rm{f}}\,\,{s}_{i,t+1}\ne {h}_{i,t+1}\\ 0, & {\rm{o}}{\rm{t}}{\rm{h}}{\rm{e}}{\rm{r}}{\rm{w}}{\rm{i}}{\rm{s}}{\rm{e}},\end{array}$$

was sufficient to achieve our objective of controlling cells to generate random, independent spike trains. Here, *S*_*i,t*_ is the state of the *i*’th cell at time *t* and *h*_*i*,*t* + 1_ is the target activity of the *i*’th cell at time *t* + 1. In order to achieve control of the *i*’th cell using DDPG, we generate a separate policy and value function for each cell, resulting in *N* policies and value functions being estimated. This approach of controlling each cell individually with a separate policy and value function is reasonable for some modern optogenetic and implanted electrode systems where stimulation is capable of overriding the influence of neighboring inputs and the targeting of single cells is possible.

We ran an experiment on a fully-actuated network of 20 SLIF cells where after a 500 episodes, the controller obtains perfect accuracy in inducing target spike trains in each cell in the network. Details regarding the initialization and parameters of this network are presented in the Methods section. One would expect the accuracy of this approach to hold for larger networks if the assumption that stimulation can always override neighboring inputs holds. Thus in this simulated setting, if not for the larger computational cost incurred by fitting and storing additional policies, controlling a small network is not more difficult than controlling a large network. Unfortunately, there are many other practical issues that make controlling a larger network much more difficult than controlling a smaller network. For example, the field of view through which neurons can be accessed in a live organism typically restricts the amount of hardware that can be used, and thus, the number of neurons that can be simultaneously simulated. One way to incorporate this limitation into our simulation involves the relaxation of the objective of inducing an independent spike train for each cell. We show in the next section how this relaxation can still lead to the induction of biologically significant states in an SLIF network.

### Under-Actuated Network Control

In general, it is impossible to induce distinct spike trains in *n* independent neurons with arbitrarily high accuracy using fewer than *n* policies. Fortunately, *in vivo* neurons in a network often display highly correlated activity patterns, suggesting that full actuation is not necessary to control many biologically meaningful states. A common model for the correlated behaviors of neurons in a network (and one compatible with the SLIF model) is based on a linear mixture model where the membrane potential of neuron *k* at time *t* is given by

11 $${V}_{k}(t)=\sum _{i}{y}_{{\rm{pre}},i}(t){w}_{i}(t)+{b}_{k}(t),$$

where *y*_pre,*i*_(*t*) is the post-synaptic potential delivered from neuron *i*, *w*_*i*_ is the synaptic weight between neurons *i* and *k*, and *b*_*k*_ is the change in *V*_*k*_ induced by neuron *k* itself (accounts for the term $\frac{-1}{{\tau }_{v}}{V}_{k}(t)+\eta {e}_{k}(t)$ in the SLIF equation)^27^. For example, correlated behaviors arise in this model system in the case of fully-connected networks of cells. In this case, if one cell in the cluster generates an action potential, then the change in the membrane potential at a subsequent timestep of all the other members of the cluster will be correlated. The situation where a network is composed of a collection of highly connected communities with sparse inter-community connections results in a covariance matrix that is approximately block-diagonal.

The study of such covariance matrices has a strong literature in the computational neuroscience community, if only indirectly, because of the popularity of methods such as Principal Component Analysis (PCA). For example, the dynamics of many large populations of neurons are known to oscillate on a low-dimensional (e.g. two or three dimensional) manifold (e.g. as shown in^16^). Results demonstrating the low-dimensional structure of the activity of large collections of neurons have been produced for a number of different model systems and conditions^22–24^. It is known that PCA achieves perfect accuracy in the recovery of communities of neurons interacting via a linear mixture in the case of a covariance matrix with perfect block-diagonal structure^28^. Though, to the best of our knowledge, no previous work exists on controlling principal component trajectories in neural systems. Some recent work exists on spectral control in large scale neural systems^15^, but it isn’t clear how this work might be applied to neural systems at different scales. We show how an underactuated system can be controlled to induce an arbitrarily structured oscillation in the phase space defined by a small number of principal components.

**Figure 2:**
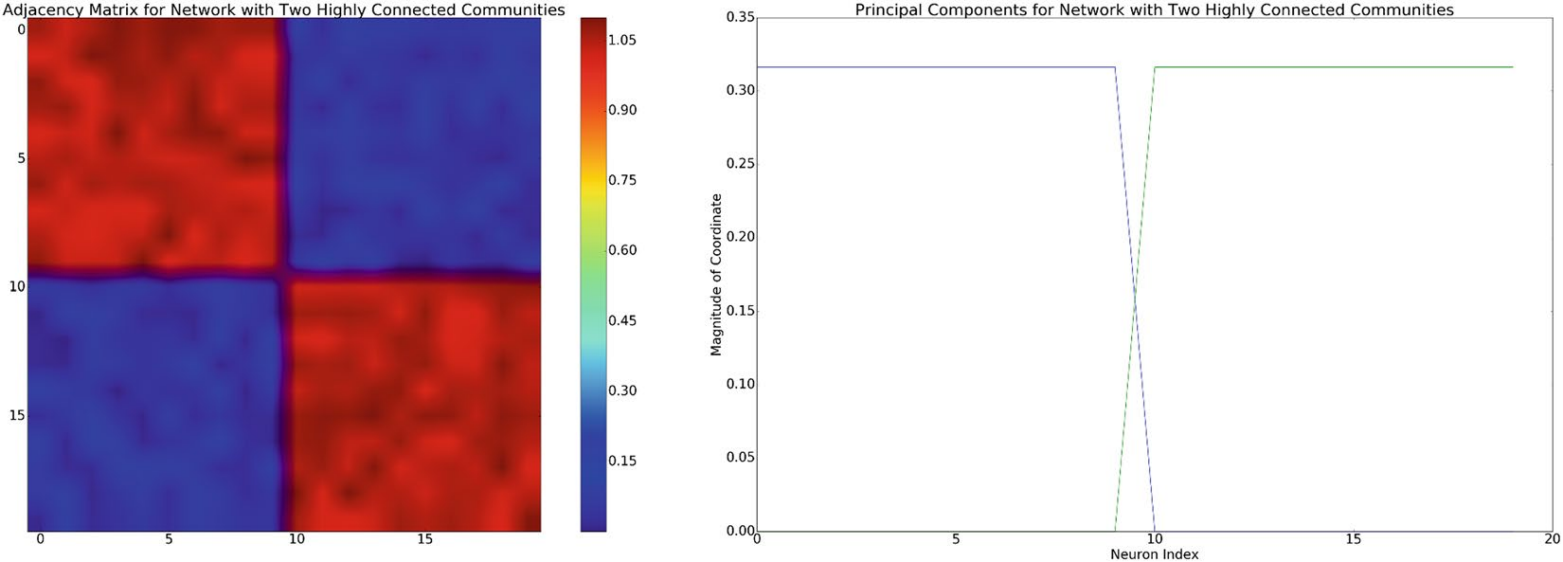
Left: example of an adjacency matrix with approximate block-diagonal structure. Assuming a linear mixture model of neuronal interactions, this network structure will induce an approximately block diagonal covariance of similar structure. Right: the principal components associated with the adjacency matrix on the left.

To do this, we construct an adjacency matrix of network connectivity with an approximate block-diagonal structure and assume recovery of the correct low-dimensional manifold (in general, this is not possible with PCA, but methods like the Treelet Transform^28^ allow the recovery of the correct manifold without requiring perfect block-diagonal structure). Examples of an adjacency matrix with two dimensional dynamics as well as the associated principal components are shown in Fig. 2. Within each community, we pick a neuron at random to receive input from a distinct policy. We then construct one and two dimensional target oscillations and attempt to reconstruct these oscillations in the phase space defined by the first one or two principal components of the neural activity.

**Figure 3:**
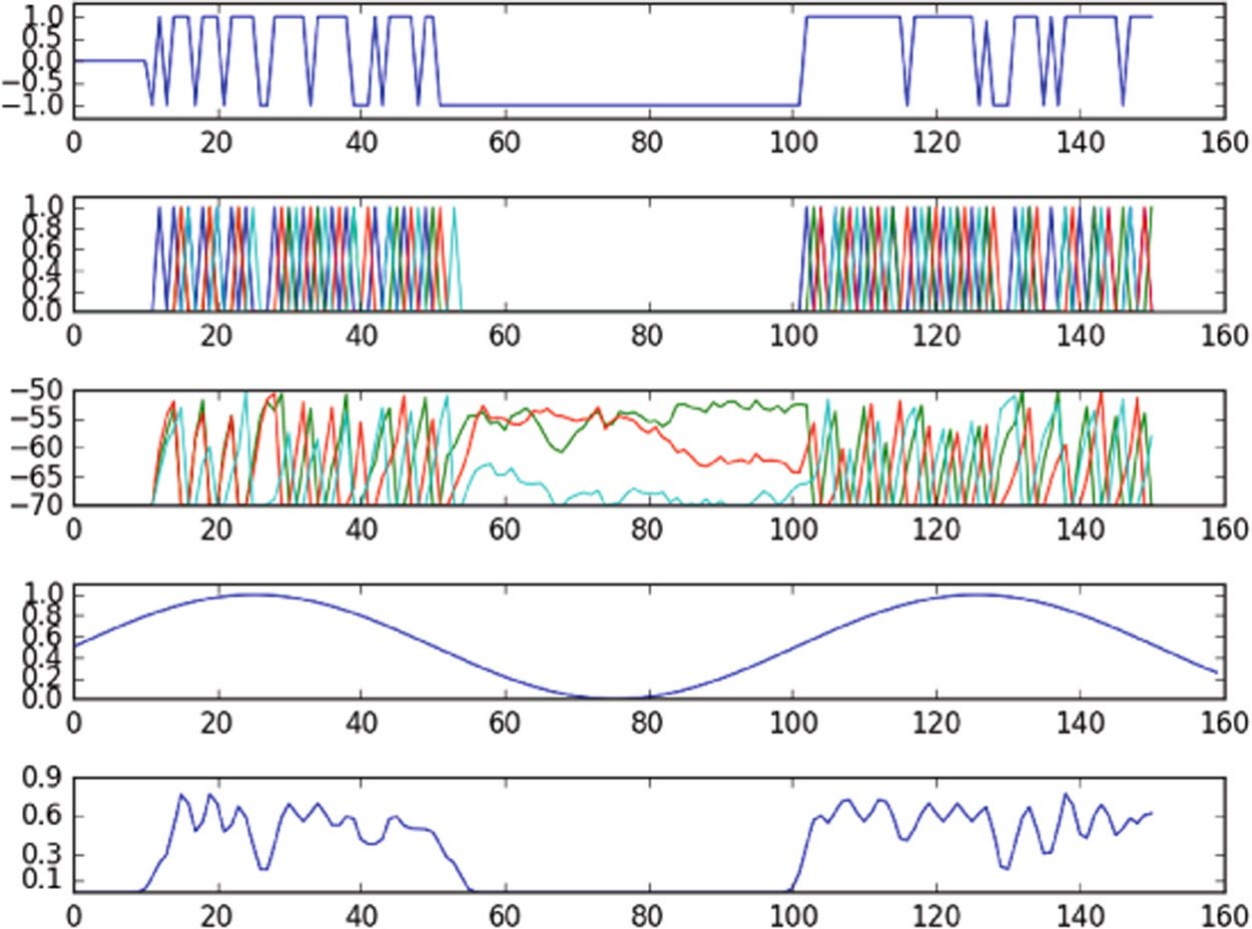
Results of the experiment controlling oscillation in the phase space defined by a single principal component. The first plot from the top is a plot of the input into the actuated cell over time; the second plot from the top is a plot of the spikes of the entire network, where different colors correspond to different cells; the third plot from the top corresponds to the membrane potential of each cell over time; the fourth from the top plot shows the target oscillation; the bottom plot shows the observed oscillation. The policy, despite delivering input to only a single cell, is able to approximately induce the target oscillation in the observed phase space.

**Figure 4:**
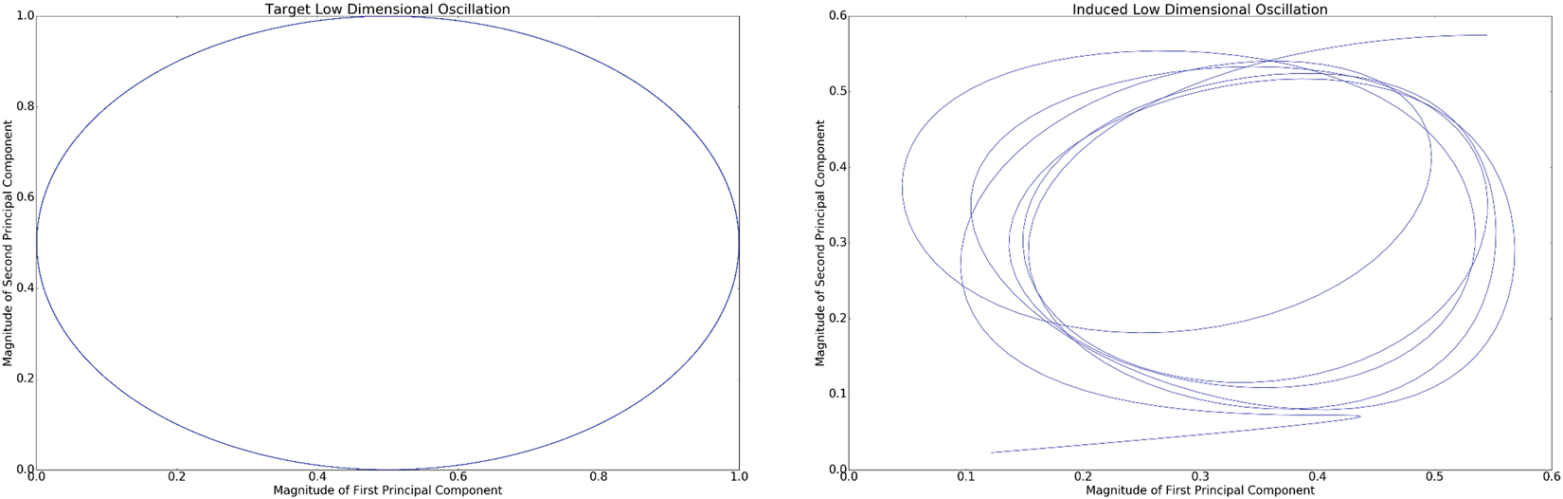
In this plot, a two-dimensional oscillation is induced in the phase-space defined by the first two principal components of the network. Left: the target oscillation. Right: the observed oscillation.

The reward function we use for accomplishing this is

12 $$r({\varphi }_{{\rm{t}}{\rm{a}}{\rm{r}}{\rm{g}}}(t),{\varphi }_{{\rm{c}}{\rm{n}}{\rm{t}}{\rm{r}}{\rm{l}}}(t))=\{\begin{array}{cc}1, & {\rm{i}}{\rm{f}}\,d({\varphi }_{{\rm{t}}{\rm{a}}{\rm{r}}{\rm{g}}}(t),{\varphi }_{{\rm{c}}{\rm{n}}{\rm{t}}{\rm{r}}{\rm{l}}}(t)) < \varepsilon \\ -1, & {\rm{o}}{\rm{t}}{\rm{h}}{\rm{e}}{\rm{r}}{\rm{w}}{\rm{i}}{\rm{s}}{\rm{e}},\end{array}$$

where *ϕ*_cntrl_(*t*) is the phase of the controlled oscillation, *ϕ*_targ_(*t*) is the phase of the target oscillation, *d*(·,·) is a distance function, and *ε* is a scalar. We define *d*(·,·) to be 0 when *ϕ*_cntrl_(*t*) is in the correct half of the unit circle at time *t* and infinity otherwise. To accomplish this, we discretize the unit circle into two halves, [0, *π*) and [*π*, 2*π*); the reward function attempts to force *ϕ*_cntrl_ to be in the same half as *ϕ*_targ_ at time *t*. We use the binary vector of action potentials at time *t* over all cells in the network to estimate *φ*_cntrl_ by first projecting it onto the first one or two principal components, then estimating the phase angle. Results from these experiments are shown in Figs 3 and 4.

We can see from these results that in the underactuated setting, while the objective is not quite as ambitious as in the fully-actuated setting (i.e. we are not able to induce an arbitrary spike-train in each cell), we can still induce physiologically meaningful states in a reasonably accurate way.

### Kuramoto Model

The Kuramoto Model (KM) characterizes the dynamics of a network of oscillators. These oscillators interact with each other over the network, and the phase of a single oscillator changes in relation to the phases of its neighboring oscillators. The KM is given by

13 $$\frac{d{\varphi }_{i}}{dt}={\omega }_{i}+\frac{K}{N}\sum _{j\mathrm{=1}}^{N}{A}_{i,j}\rho ({\varphi }_{j}-{\varphi }_{i}),$$

where *N* is the number of oscillators, *K* is the coupling strength, *A*_*i*,*j*_ is the edge weight between the *i*’th and *j*’th oscillators, *ϕ*_*i*_ is the phase of the *i*’th oscillator, *ω*_*i*_ is the natural frequency of the *i*’th oscillator, and *ρ* is a non-linear function characterizing the influence of neighboring oscillators on the *i*’th oscillator. In the original KM model, sin(⋅) was used as the non-linearity. All values of model parameters used to generate the results shown in this paper are given in the Methods section.

We adopt a recent control strategy for this model which was originally introduced for disrupting the synchronizing effects of the rightmost term in the KM (e.g.^29,30^). It is known that for large *K*, the network of oscillators synchronize over time. An analytical solution for desynchronization of the oscillators is developed in^30^. That framework takes the form

14 $$\frac{d{\varphi }_{i}}{dt}={\omega }_{i}+\frac{K}{N}\sum _{j\mathrm{=1}}^{N}{A}_{i,j}\rho ({\varphi }_{j}-{\varphi }_{i})+\frac{d{\varphi }_{i}^{{\rm{ctrl}}}}{dt},$$

where ${\varphi }_{i}^{{\rm{ctrl}}}$ is an additive control term. With DDPG, rather than focusing on the popular problem of desynchronizing oscillators bound to synchronize, we address the problem of inducing synchrony in networks that will not synchronize on their own (i.e. with small *K*). To relate the controlled KM presented above with the DRL framework, consider a forward Euler discretization of the above ODE:

15 $$\frac{{\varphi }_{i,t}-{\varphi }_{i,t-1}}{{\rm{\Delta }}t}={\omega }_{i}+\frac{K}{N}\sum _{j\mathrm{=1}}^{N}{A}_{i,j}\rho ({\varphi }_{j,t-1}-{\varphi }_{i,t-1})+\frac{{\varphi }_{i,t}^{{\rm{ctrl}}}-{\varphi }_{i,t-1}^{{\rm{ctrl}}}}{{\rm{\Delta }}t}\mathrm{.}$$

where ${\varphi }_{i,t}^{{\rm{ctrl}}}$ is the input helping induce the transition to *ϕ*_*i*,*t* + 1_. Solving for ${\varphi }_{i,t}^{{\rm{ctrl}}}$, we obtain

16 $${\varphi }_{i,t}^{{\rm{ctrl}}}=-\,{\rm{\Delta }}t({\omega }_{i}+\frac{K}{N}\sum _{j\mathrm{=1}}^{N}{A}_{i,j}\rho ({\varphi }_{j,t-1}-{\varphi }_{i,t-1}))+{\varphi }_{i,t-1}^{{\rm{ctrl}}}+{\varphi }_{i,t}-{\varphi }_{i,t-1}\mathrm{.}$$

Here ${\varphi }_{i,t}^{{\rm{ctrl}}}$ can be considered a policy, ${\mu }_{i}({a}_{t}^{i}|{s}_{t})$, where the state, *s*_*t*_, is $({\varphi }_{i,t},{\varphi }_{i,t-1}^{{\rm{ctrl}}},{\varphi }_{\mathrm{1,}t-1},\ldots ,{\varphi }_{N,t-1})$. Using DDPG, we can extend this idea so that ${\mu }_{i}({a}_{t}^{i}|{s}_{t},{\theta }^{{\mu }_{i}})$ can be defined using a deep neural network with parameters ${\theta }^{{\mu }_{i}}$.

We apply this approach to the problem of synchronizing a network of weakly coupled oscillators by entrainment to a reference oscillator. This problem has recently been considered in^7,8^. An important result from this work is that the bound on the coupling strength below which synchronization no longer occurs, ${K}_{c}^{{\rm{unf}}}$, is lowered by forcing input to *K*_*c*_. If *p*(*ω*) is the distribution from which the natural frequencies are drawn and it is unimodal and symmetric about 0, then ${K}_{c}^{{\rm{unf}}}=\frac{2}{\pi p\mathrm{(0)}}$ as *N* → ∞. In contrast, in the presence of forcing, the minimum coupling strength required for synchronization is reduced to ${K}_{c}=\frac{2}{\pi }$. Below this coupling strength, while entrainment imposes the reference frequency on each oscillator in a network, the phases achieve nominal synchronization (between 0.0 and 0.2)^7^. To demonstrate the utility of DDPG for model-free control of the Kuramoto model, we pick a coupling strength below *K*_*c*_ (*K* = 0.1) and show that the synchronization achieved is significantly higher than this (about what one would expect for a coupling strength of slightly greater than $\frac{2}{\pi }$ using the method in^7,8^).

We define the state space to be the set of phases of all oscillators over a 40 timestep history, along with the adjacency matrix of the network. The reward function was defined to be

17 $${r}_{i}({s}_{t},{a}_{t}^{i})=\frac{q+\varepsilon {q^{\prime} }_{i}+\eta ||{a}_{t}^{i}{||}_{1}}{2+\varepsilon },$$

where *ε*, *η* ∈ [0, 1] and *q* and *q*′_*i*_ are defined to be the order of synchronization. This quantity is given by

18 $$q{e}^{i\psi }=\frac{1}{N}\sum _{j\mathrm{=1}}^{N}{e}^{i{\varphi }_{j}},$$

where *ψ* corresponds to the average phase of the oscilators. The difference between *q* and *q*′ is that *q* corresponds to the synchronization of all *N* oscillators, while *q*′_*i*_ is defined to be the synchronization of the *i*’th oscillator with respect to a reference oscillator. It was observed that without this term in the reward function, the policy regularly failed to induce global synchronization. To stabilize the controller, one oscillator in the network was chosen at random to be the reference, though an external oscillator might also be used. Regularization by the norm of the action was also included, to encourage more reliable exploration of the state-action space. Specifically, without this regularization, the controller would favor large actions, even though these actions were rarely optimal. This regularization was thus included to encourage more thorough exploration of the state-action space.

**Figure 5:**
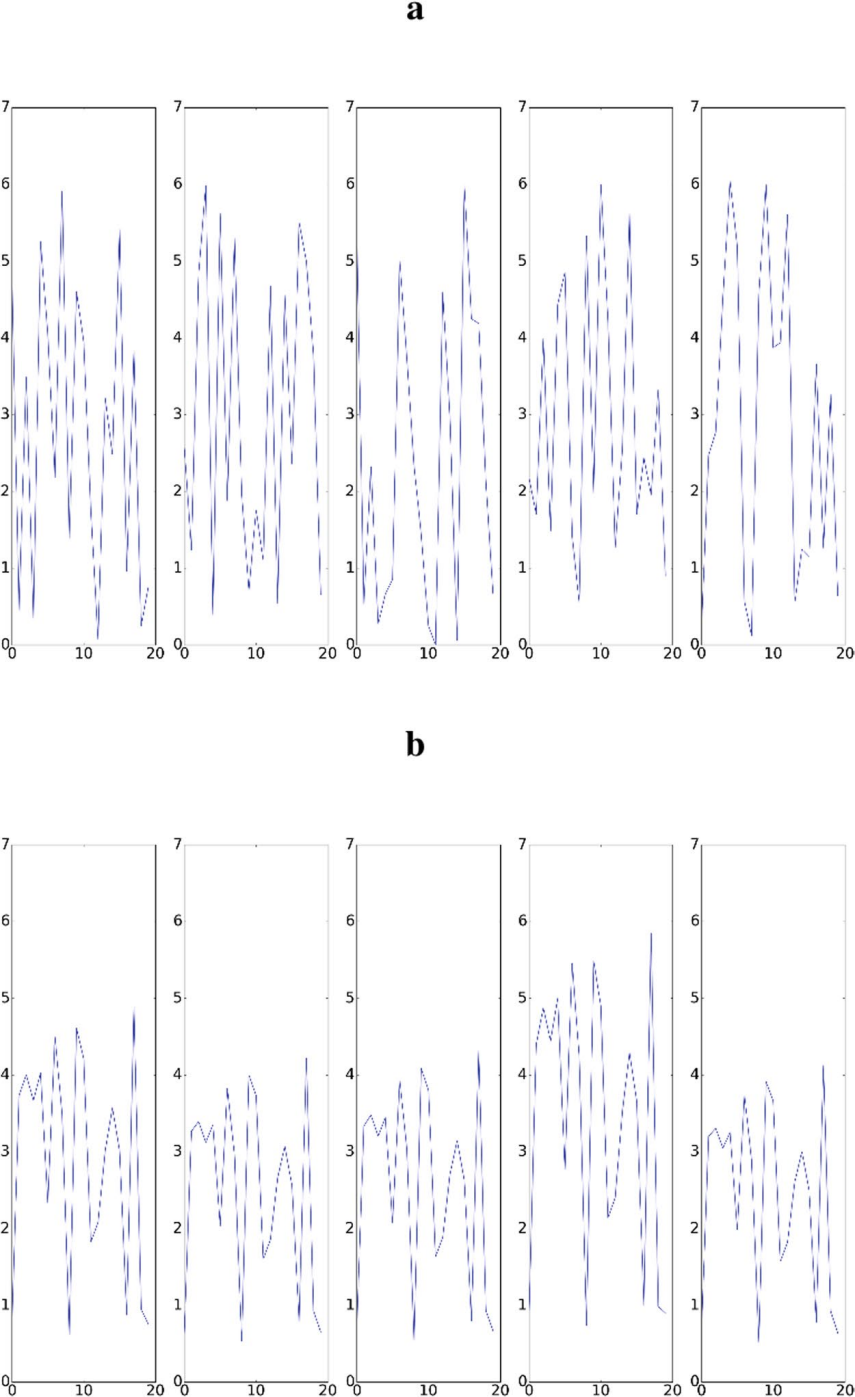
Examples demonstrating induction of synchronization by controller for weakly coupled oscillators. For all plots, the vertical axis is the phase and the horizontal axis is timestep. For both sections a and b, five plots are included generated from five randomly selected oscillators from the network of 20 oscillators. (**a**) Phases for oscillators before global synchronization has been induced by the controller. (**b**) Phases for oscillators after global synchronization has been induced by the controller. This level of synchronization can be observed after a few hundred training episodes have been observed.

**Figure 6:**
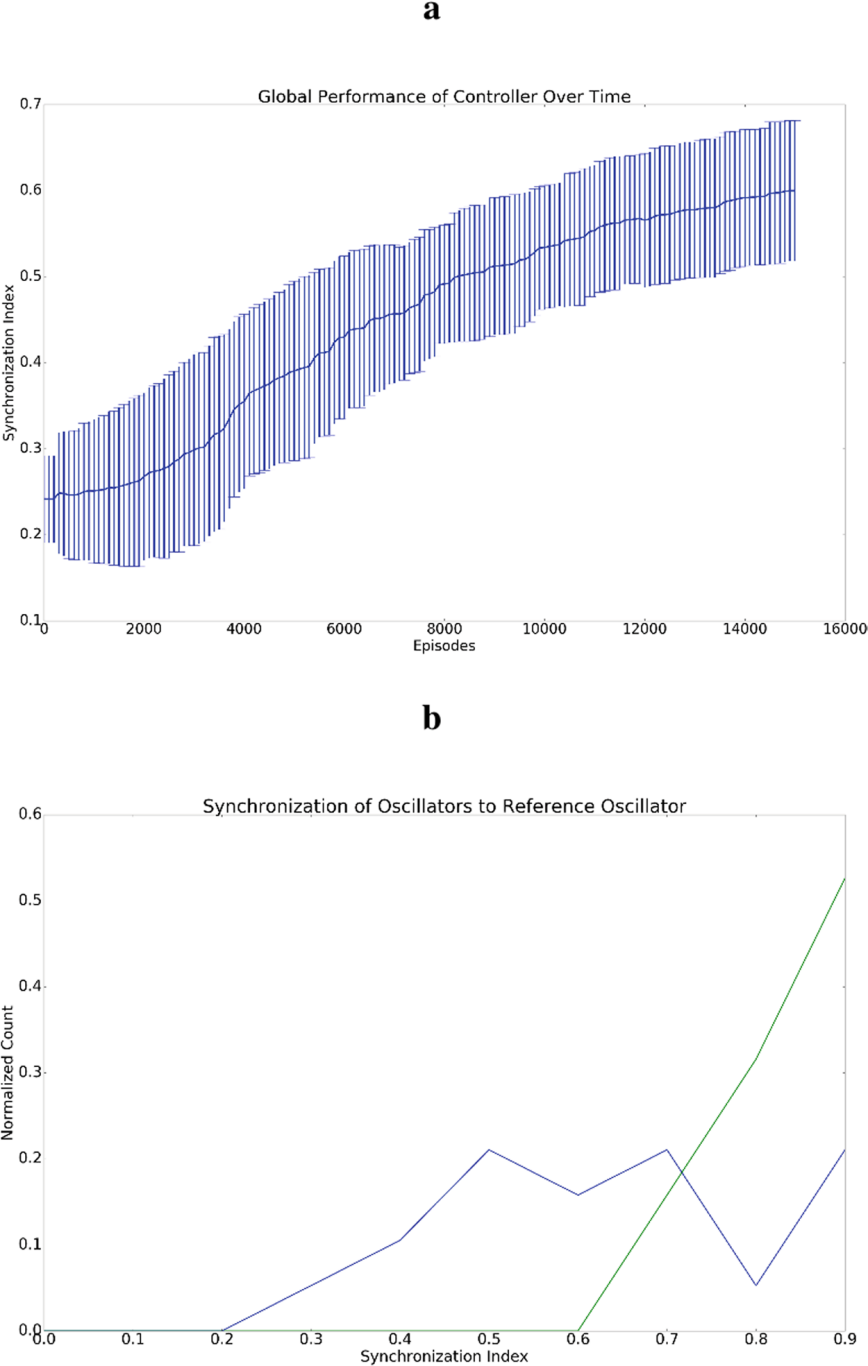
Summary results of 10 synchronization experiments. (**a**) Depicts the mean and standard deviation of the global synchronization, (i.e. *q* from equation 16), against the number of training periods of the controller. (**b**) Shows histograms demonstrating the synchronization level of all network oscillators with the reference oscillator (i.e. *q*_*i*_ from equation 16). That is, a point on either the blue or green curves demonstrates the probability of having a given value for qi. The blue histogram shows counts before training while the green histogram shows counts after training. The average synchronization with the reference, *q*_*i*_, is much higher than global synchronization, *q*, which is explained by the fact that synchronization with the reference is easier to induce than global synchronization.

It can be seen from Figs 5 and 6 that the network eventually synchronizes. To understand this result, it may help to view the reward function as a shaped reward. This interpretation follows from the fact that synchronization of each oscillator in the network with a single reference oscillator is far easier than inducing global synchronization of the entire network. This point is emphasized by Fig. 6 which shows that almost all oscillators in the network achieve very high synchronization with the reference oscillator, and in general, the average synchronization with the reference is much higher than the global synchronization of the entire network. That first synchronizing with the reference makes global synchronization easier to obtain follows from the fact that the size of the state space to be explored is reduced. So, rather than having to tune frequencies and phases of the oscillators in the network, synchronization with the reference frequency-locks all oscillators and thus, global synchrony can be induced by adjusting their respective phases.

## Discussion

The model-free approaches to control of neural systems presented here suggest that deep reinforcement learning has potential for application to this area. We show how the engineering problem is transformed from one that focuses on the design of appropriate system dynamics and the control of these models, to the design of good reward functions that allow for accurate and tractable optimization of the respective objectives. In the case of inducing target spike trains or latent trajectories in the SLIF model, the rewards were able to directly represent these objectives. The problem of inducing synchronization in a weakly coupled network of oscillators required the introduction of a shaped reward to allow for robust synchronization of the network. The problem of designing appropriately shaped rewards is a significant problem, not just in the application of DDPG (e.g. as in^31^), but for model-free reinforcement learning in general^32^. In this work, we manually design the reward functions used to estimate optimal policies.

Arguably, this is not the most efficient way to find an optimal policy and in fact, several methods exist for combining model-free reinforcement learning with inverse reinforcement learning (IRL) algorithms, which are used to infer a reward function given state-action pairs sampled from an optimal policy^33–36^. And indeed, this is a interesting direction for future work. We attempted with this work to find a compromise between automating the discovery of good policies and allowing for interpretability of the actions of the learner. IRL presents a powerful set of tools with which policies can be learned to perform complex objectives, but for the first application of deep reinforcement learning to biological systems, we felt it best to ensure that the problems being solved had clear, interpretable control objectives. The ultimate goal of this work is to apply these algorithms to human biology and in all such work, there is necessarily a balance between technological advance and safety.

Ideally, we would be able to take an optimal policy and deduce either an analytical form or empirical results explaining its performance. In fact, developing methods to allow deep neural networks to “explain themselves” is an active area of research in the machine learning and statistics communities. These methods are being proposed in response to the classical notion put forth famously by Richard Feynman who said, “What I cannot create, I do not understand”. This philosophy would suggest to replace mysterious, poorly understood parts of deep reinforcement learning with manually constructed models (e.g. in a similar vein as what is done to improve the sample-complexity of model-free methods by incorporating manually designed components^18^). Alternatively, attempts have been made to leave a black-box machine learning algorithm intact and attempt to better understand it. For example, a method using influence functions was recently developed to yield insight into how deep neural networks work when used for supervised learning^37^; extending methods like that in^37^ for problems in reinforcement learning is another interesting direction for future work. Further extensions to alternating IRL/policy optimization solvers (e.g. as in^35^) is an even longer term goal.

There are other factors to consider in the application of deep reinforcement learning algorithms to biological systems in addition to the ability of humans to understand them. For example, our work assumes full observability of the system state. Relaxing this assumption to partial observability is required in some applications (requiring the use of methods such as^38^): the manner in which the uncertainty induced by partial observability interacts with modeling uncertainty is an important problem for applications to biology. The off-policy nature of DDPG allows for a reduced number of samples from the policy during learning and thus, the use of experience replay during learning. This can reduce the number of times a controller would need to interact with an actual brain in order to fit a policy. Another approach for improving the efficiency of exploration was proposed in^31^, where the authors show that data generation and resampling efficiency can be improved relative to the number of parameter updates. For complex objectives, it may be helpful to initialize the search for an optimal policy from states that have achieved a partial reward. Both methods help to accelerate discovery of optimal policies for complex control objectives.

## Conclusion

We have presented a model-free control strategy for the control of neural systems at multiple scales. We demonstrated the use of this strategy on a large-scale system (the Kuramoto Model) and a small-scale system (a network of Stochastic, Leaky Integrate and Fire neurons). We showed that this approach can be used to solve difficult, unsolved control problems including the induction of latent trajectories in the network of SLIF cells as well as the synchronization of weakly coupled Kuramoto oscillators. The success of this approach has been demonstrated in simulation, but we believe that it has potential for application to physical systems. A number of the extant issues with model-free deep reinforcement learning as applied to biological systems have been discussed and hopefully further work to address these issues will be forthcoming to accelerate progress in this exciting new application area.

## Methods

### Deep Neural Network Parameters

For the approximation of the Q-function as well as the policy and target networks, deep networks with two hidden layers were used. For the network of SLIF neurons, the first hidden layer had 400 units and the second had 300 units, while for the KM, the first hidden layer had 1200 units and the second had 1000. A value of $\tau =0.001$ was used in the exponential moving average between updated network parameters and past network parameters. ADAM^39^ was used for stochastic gradient descent to perform parameter updates. The same learning rate was used for fitting the actor and critic networks for both model systems. For control of the network of SLIF neurons, a learning rate of 0.01 was used, while for control of the KM, a learning rate of 0.0001 was used. These learning rates were chosen based on the rates used in the original DDPG paper (KM control) or slightly modifying these values (SLIF control). The learning rate was increased for SLIF control because it was observed that if this rate were too low, the optimal stimulation strength was never reached and reliable spiking behavior wasn’t induced in any of the cells. Mini-batch stochastic gradient descent was performed with a batch size of 32 4-tuples, where each 4-tuple contained (*s*_*t*_, *a*_*t*_, *r*(*s*_*t*_, *a*_*t*_), *s*_*t* + 1_), for some time *t*. The discounted rate of future returns used was *γ* = 0.99.

### SLIF Parameters

We used *C* = 10.0, *τ*_*v*_ = 15.0, a resting membrane potential of −70.0, a depolarization threshold of −50.0, a refractory period of length 0., *τ*_*s*_ = 1.0, Δ*t* = 1.0, $\bar{g}$ = 0.01, $\eta =\sqrt{2}$, and *E*_*syn*_ = 70.0^17,18^. All simulations were begun with cells at the resting membrane potential. Before the controller delivered input into the system in both training and testing instances, the network was reinitialized to resting membrane potential. For the fully-actuated example, network adjacency matrices were initialized with directed edges whose weights were drawn from a uniform distribution over [0, 1]. This matrix was made symmetric for the under-actuated example. Simulations were run on networks as large as 40 cells for the fully-actuated example; 4 neuron networks were used for the 1-D under-actuated example and 32 neuron networks were used for the 2-D under-actuated example. For the fully-actuated example, the state consisted of a 10 timestep spike history concatenated with a 10 timestep lookahead into the target spike train. Similarly, for the under-actuated example, a 10 timestep spike history was concatenated with a 10 timestep lookahead to construct the state. Since the projection of the binary vector of all neuron spikes onto a principal component effectively gives an instantaneous firing rate of all neurons in a given community, the lookahead in this case consisted of sampled spiking activity of the controlled cell at the target rate.

### KM Parameters

The controller was tested on a network of 20 oscillators, each initialized to a phase (*ϕ*_*i*_) drawn uniformly at random from [0, 2*π*], with natural frequencies (*ω*_*i*_) drawn from *N*(0, 10), and edge weights (*A*_*i*,*j*_) drawn from a uniform distribution over [0, 1]. For our experiments, we chose *K* = 0.1. *ε*, *η* were 0.1 and 1.0 respectively.

### Code Availability

All code required to replicate the results presented in this paper will be made publicly available on Github after publication.
